# Persistent Zika Virus Infection Associated with Early Fetal Demise: A Case Report

**DOI:** 10.4236/ojog.2019.95069

**Published:** 2019-05

**Authors:** Janice Pérez-Padilla, Gabriela Paz-Bailey, Dana Meaney-Delman, Kate Doyle, Joy Gary, Dania M. Rodriguez, Julu Bhatnagar, Nicole M. Pérez-Rodriguez, Sara Montalvo, Luisa Alvarado, Tyler M. Sharp

**Affiliations:** 1 Centers for Disease Control and Prevention (CDC), Dengue Branch, San Juan, Puerto Rico; 2 CDC, National Center for Emerging and Zoonotic Infectious Diseases, Atlanta, GA, USA; 3 CDC, Division of HIV/AIDS Prevention, Atlanta, GA, USA; 4 CDC, Infectious Disease Pathology Branch, Atlanta, GA, USA; 5 Ponce Health Sciences University/Saint Luke’s Episcopal Hospital, Ponce, Puerto Rico

**Keywords:** Zika, Pregnancy Outcomes, ZIKV Persistence

## Abstract

**Background:**

Infection with Zika virus (ZIKV) during pregnancy is known to cause birth defects and could also be linked to pregnancy loss.

**Case:**

A pregnant woman in Puerto Rico contracted ZIKV at 16 weeks gestation. ZIKV RNA persisted in serum from her initial test at 16 weeks through 24 weeks gestation, when fetal demise occurred, and was detected in placental tissue.

**Conclusion:**

Prolonged detection of ZIKV RNA in maternal serum was associated with ZIKV RNA detection in the placenta of a patient who experienced fetal demise. While detection of placenta ZIKV RNA does not establish that ZIKV conclusively caused the demise, these findings support emerging evidence that the placenta may serve as a reservoir for ZIKV, which may be associated with prolonged detection of ZIKV RNA in serum.

## 1. Introduction

Zika virus (ZIKV) is primarily transmitted by *Aedes* species mosquitoes, and is closely related to dengue, West Nile, and yellow fever viruses. Although most ZIKV infections are asymptomatic, those who do develop disease experience a mild illness characterized by rash, fever, arthralgia, myalgia, and/or non-purulent conjunctivitis. When first isolated in 1954 [1], ZIKV was reported to cause a mild illness without observed severe manifestations. Few human cases were identified over the subsequent 50 years until a ZIKV outbreak occurred in 2007 in the Pacific island nation of Yap, Federated States of Micronesia [2]. Subsequent outbreaks occurred in French Polynesia during 2012–2013 [3] and in 2015 ZIKV spread to Brazil and throughout the Americas and the Caribbean [4] [5] [6]. After the emergence of ZIKV in Brazil, it was found that ZIKV causes birth defects in children congenitally infected and has been associated with other adverse pregnancy outcomes [7] [8] [9] [10]. Prolonged detection of ZIKV ribonucleic acid (RNA) has been observed among pregnant women [11]; however, the clinical significance of this finding has not been firmly established. Some have suggested that prolonged detection of viral RNA may be a marker of fetal infection and adverse pregnancy outcomes [7]. ZIKV infection early in pregnancy has been associated with prolonged detection of ZIKV RNA in serum and infectious virus was isolated from the mother’s placenta tissue and maternal serum in a pregnancy loss [12]. Case series of women infected with ZIKV during the first and second trimester demonstrated the presence of ZIKV in placental and fetal tissue, suggesting that ZIKV RNA can persist in these tissues [13] [14] [15].

The first locally acquired case of ZIKV infection in Puerto Rico was reported in December 2015. Patients with symptomatic ZIKV disease were identified through passive surveillance, and in early 2016 the Puerto Rico Department of Health (PRDH), with assistance from the Centers for Disease Control and Prevention (CDC), implemented routine screening of pregnant women with RT-PCR to detect viral RNA and ELISA testing to detect anti-ZIKV IgM antibodies. By the end of 2017, over 4000 pregnant women were diagnosed with laboratory evidence of possible ZIKV infection [16].

Detection of ZIKV RNA by RT-PCR is possible in various body fluids, including serum, urine, saliva, semen, vaginal secretions, cerebrospinal fluid, and amniotic fluid [17] [18] [19] [20]. Clearance of ZIKV nucleic acid from serum is expected to occur in most non-pregnant individuals within two months of infection [3] [20]. However, several studies indicate that ZIKV RNA may be detectable for longer periods in pregnant women [11] [21]. Hypotheses as to why pregnant women may experience prolonged detection of ZIKV RNA include replication of ZIKV in the placenta, specifically in placental trophoblasts [22] [23] and Hofbauer cells (HBC) [24]. This finding led to the conclusion that transmission of ZIKV from mother to fetus is likely trans-placental, rather than during parturition [13] [22] [24], and suggests that viral replication in the placenta may precede fetal infection [13]. The mechanism and correlation of viral persistence in the placenta and detection in human serum is not known. This case report provides additional clinical information adding to the growing body of evidence that will elucidate the pathologic and clinical findings associated with maternal and congenital ZIKV infection.

## 2. Case

In May 2016, a 38 year-old nulliparous woman at approximately 16 weeks’ gestation sought care for a one-day history of fever, rash, myalgia, arthralgia, and headache. Her only significant medical history was well-controlled high blood pressure. Diagnostic testing for toxoplasmosis, rubella, cytomegalovirus, herpes simplex virus, and human immunodeficiency virus (HIV) (*i.e*., TORCH) performed at 10 weeks’ gestation was negative. At the time of her visit, the patient agreed to participate in an ongoing study of acute febrile illness, which collected blood, urine, and nasopharyngeal specimens for diagnostic testing [25].

ZIKV RNA was detected in serum by RT-PCR one day after symptom onset; serum IgM testing and urine RT-PCR were both negative. The patient was enrolled in the Zika virus Persistence study [20], for which serum, whole blood, urine, saliva, and vaginal secretions were collected weekly for 1 month and subsequently every 2 weeks thereafter. Collected serum specimens were tested for ZIKV, dengue virus, and chikungunya virus nucleic acid by RT-PCR [26], and for anti-ZIKV IgM and anti-dengue virus IgM and IgG antibodies by ELISA [20].

**Figure 1.**
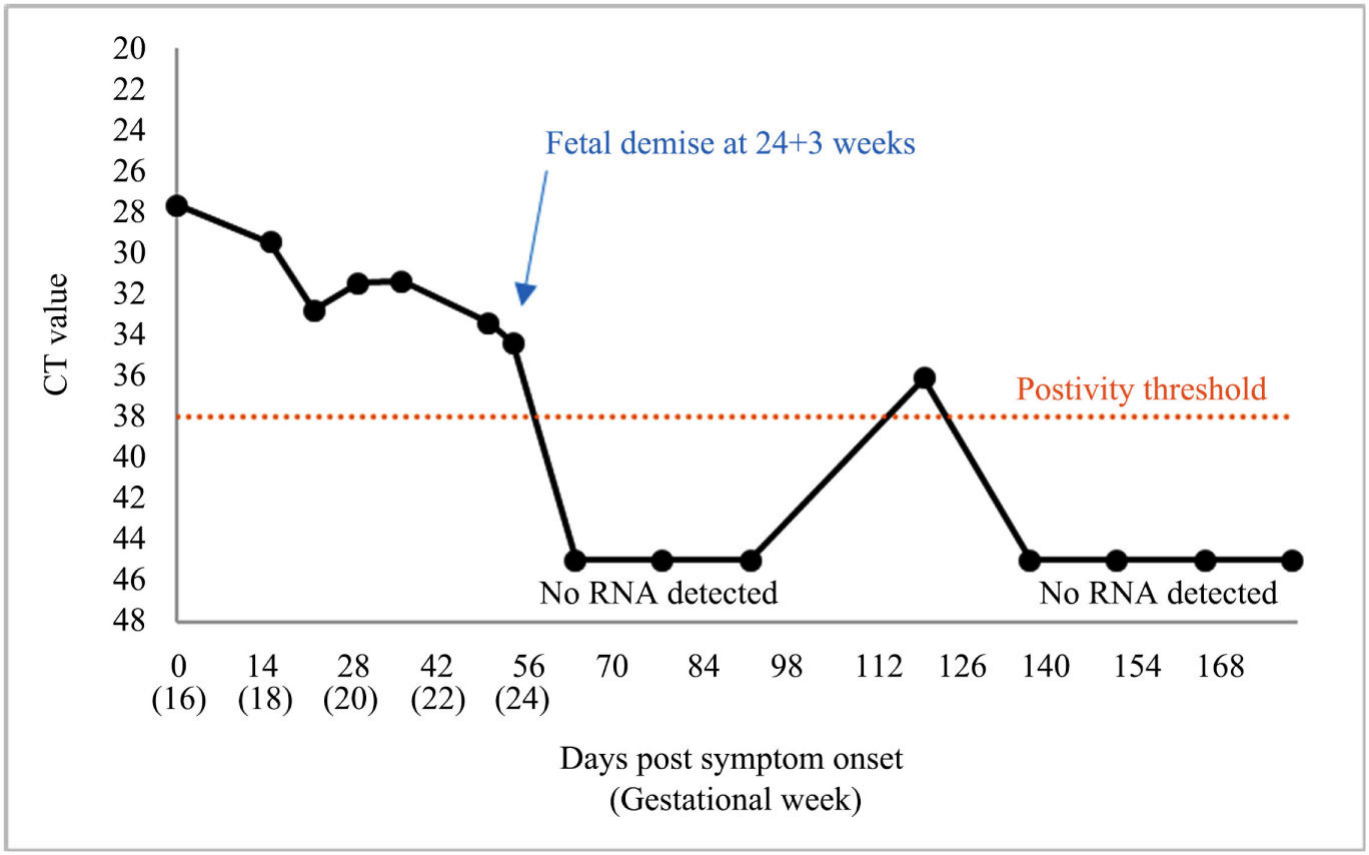
Detection of Zika virus nucleic acid in serum specimens from an infected pregnant woman, Puerto Rico, 2016.

ZIKV RNA was detected in serum on days 1, 15, 22, 29, 36, 50 and 54 post illness onset (*i.e*., 18 – 24 weeks’ gestation) (Figure 1). The patient was referred to a maternal-fetal medicine (MFM) specialist at 20 weeks’ gestation due to a positive quadruple screen test, 1:8 suggestive of Down syndrome, and a positive test for ZIKV infection. At approximately 24 weeks’ gestation, a level II ultrasound demonstrated generalized edema predominantly localized to the upper portion of the fetus’ body, a small amount of ascites, a single umbilical artery, and the absence of cardiac activity consistent with fetal demise. The patient was admitted to a hospital the following day for labor induction. TORCH testing was repeated, testing was performed for Lupusanticoagulant, and urine and serum were screened for drug and toxins, all of which were negative. The fetus was delivered vaginally at 24 weeks’ gestation in breech position approximately 8 hours after the initiation of labor induction. No cardiac or respiratory activity was present in the fetus at birth consistent with the diagnosis of fetal demise. Fetal weight was 3,289 grams. The fetus was edematous and macerated, and skin desquamation was present in the upper and lower extremities. The abdomen had skin discoloration suggestive of possible hematoma. Thinning of the umbilical cord at insertion site was noted. No dysmorphic features or neural tube defects were observed, and no clinical stigmata of Down’s syndrome were present. A retained placenta requiring a dilation and curettage complicated the mother’s clinical course. She recovered satisfactorily and was discharged home the following day. Parental consent was not provided for fetal autopsy; however, consent was given to examine the umbilical cord and placenta.

The placenta weighed 213 grams and measured 12 × 11.3 × 2.8 cm. The fetal and maternal membranes were well vascularized and the umbilical cord was inserted at the margin, measuring 23 × 1 cm, with two blood vessels present. The maternal surface had complete cotyledons, and transverse sections revealed spongy tan parenchyma. Upon microscopic evaluation, the placenta had patchy villous edema. Karyorrhectic debris was present around vessels within edematous villi, and larger sclerotic, avascular villi often had associated perivillous fibrin deposition. Stippled, deeply-basophilic calcifications were scattered within multiple villi. Multifocal aggregates of plasma cells were present within the decidua. Histologic findings were nonspecific, consistent with findings seen with intrauterine fetal demise and/or circulatory issues within the placenta, though the presence of plasma cells and other mononuclear cells within the decidua has been described as a sign of chronic antigenic stimulation [27]. ZIKV RNA was detected by RT-PCR in a specimen of placenta; however, ZIKV antigen was not detected by immunohistochemistry.

The patient was followed as part the Zika Virus Persistence study protocol. RT-PCR testing did not detect ZIKV RNA at 64, 78, and 92 days post-illness onset (Figure 1). On day 120 after illness onset, ZIKV RNA was detected at low levels in a serum specimen. All serum, urine, saliva and vaginal secretions collected thereafter were negative by RT-PCR. Anti-ZIKV IgM antibody was first detected in serum on day 15 post-illness onset and was last detected on day 78 post-illness onset (Table 1). The same specimens were also tested by anti-DENV IgM ELISA, and all were negative. Anti-DENV IgG antibody was detected by ELISA in the specimen collected on the first day of acute febrile illness (week 16 gestation), demonstrating previous DENV infection. Further analysis of neutralizing antibodies by plaque reduction neutralization test (PRNT) on the same specimen [28] [29] demonstrated high titers against DENV−2, low titers to DENV−1, −3, and −4, and no titers against ZIKV, which together suggested prior infection with DENV−2. Specimens collected in the weeks following the initial infection demonstrated a robust neutralizing antibody response to ZIKV, including greater than 4-fold increase in titers against all DENVs, consistent with antibody response to secondary flavivirus infection [30].

## 3. Discussion

ZIKV infection has been shown to cause birth defects among children congenitally infected and is linked to other abnormal pregnancy outcomes [31], including pregnancy loss [12] [13] [14] [15]. In this case report, ZIKV RNA was detectable throughout pregnancy and only became non-detectable by RT-PCR following delivery of the fetal demise, and ZIKV nucleic acid was detected in placental tissue. These findings support the notion that the placenta is a potential site of infection and may serve as a reservoir of ZIKV RNA [13] [15] [22] [24] [32]. As an immunologically privileged site, it may take longer for maternal clearance of ZIKV RNA due to the down regulation of the maternal-fetal immune interface. The immune changes during pregnancy, which can allow pathogens to avoid clearance and be transmitted from the mother to the fetus, are not well understood but may involve the production of inhibitory cytokines (e.g., IL-35) by placental trophoblasts [33].

To date, the factors that predispose some pregnant women with ZIKV infection to adverse pregnancy outcomes and why others clear the infection without an effect on the fetus is not clear. Factors that may affect the likelihood of ad verse pregnancy outcomes are the timing of infection, viral load, and the maternal immune response. Infection during certain critical time points during pregnancy may allow for a blunted or heightened maternal immune response. Placental blood flow, initiated at approximately gestational week 10, may also affect risk of fetal infection and adverse outcomes [34]. This patient had evidence of acute infection at 16 weeks gestation, a time at which the maternal infection may already have resulted in fetal infection. Fetal immunity is just beginning to develop at this early stage of gestation; hence, clearance of the infection is reliant on the maternal immune response.

Interestingly, testing of maternal serum was repeatedly negative by RT-PCR between 64 and 92 days post-onset of illness; however at day 120, a small amount of ZIKV RNA was detected. The clinical significance of this finding is unclear, and false positive test results have been reported [35]. Of note, intermittent detection of ZIKV RNA has been reported in both pregnant and non-pregnant individuals [11] [20] [36]. Also, the positive RT-PCR on day 120 may not have represented active viral replication [37]. Similar observations of intermittent shedding of viral RNA have been observed for ebolavirus [38], HIV [39], and other flaviviruses (*i.e*., Japanese encephalitis virus, West Nile virus, and tick-borne encephalitis virus) [37]. Therefore, the isolated detection on day 120 after symptom onset could also suggest that the placenta or another tissue may have acted as a reservoir of and released fragments of viral nucleic acid into the serum.

This case demonstrates the importance of monitoring women infected with ZIKV during pregnancy and the need to counsel women of the potential risks of ZIKV infection during pregnancy. Limited data are available regarding the prolonged detection of ZIKV RNA among pregnant women; therefore assessing the association of pregnancy outcomes with prolonged detection of RNA will be important. Further, research is needed to conclusively determine if the placenta does indeed serve as a reservoir for ZIKV replication, and the mechanism by which the placenta regulates maternal-fetal transmission of infection. Last, establishing whether prolonged detection of ZIKV RNA in serum is a predictor of fetal demise or other adverse pregnancy outcomes will be important. Immunocompromised mice infected with ZIKV early in pregnancy demonstrate placental damage and fetal demise, and ZIKV RNA was detected in the placenta at levels ~1000-fold higher than in maternal serum [23]. If the same occurs in humans, we could infer that high levels of ZIKV RNA in serum may have the potential to serve as a marker for placental infection and higher placenta viral loads.

Repeat testing of women with an established diagnosis of ZIKV infection is not currently recommended for monitoring pregnant women exposed to ZIKV since the clinical significance of the findings is unknown [40]. Additional research is needed to determine the clinical value of such repeat testing, and to assess if repeat testing by RT-PCR in ZIKV-positive pregnant women can predict adverse pregnancy or fetal outcomes or pregnancy loss.

## 4. Conclusion

Upon physical examination of the fetus, no dysmorphic features were observed and no clinical stigmata of Down’s syndrome were present. However, since autopsy of the fetus could not be performed due to lack of parental consent, Down syndrome could not be completely ruled out. Nonetheless, more research is needed to establish what the risk of pregnancy loss is for women infected with ZIKV during pregnancy and to understand the role of persistent viremia in these outcomes.

## Figures and Tables

**Table 1.** Detection of anti-Zika virus IgM antibody and neutralizing antibody titers against Zika virus and the four dengue virus types in a pregnant woman infected with Zika virus, Puerto Rico, 2016.

		Plaque Reduction Neutralization Test (PRNT)				
Days Post Onset of Symptoms	Anti-ZIKV IgM ELISA	ZIKV	DENV-1	DENV-2	DENV-3	DENV-4
0	Neg	<10	20	640	40	40
15	Pos	5120	2560	>20,480	2560	320
22	Pos	5120	2560	>20,480	2560	160
29	Pos	5120	1280	≥20,480	640	160
36	Pos	5120	320	20,480	640	320
50	Pos	5120	640	20,480	640	160
54	ND	10,240	320	10,240	640	160
64	Pos	ND	ND	ND	ND	ND
78	Pos	ND	ND	ND	ND	ND
92	Neg	ND	ND	ND	ND	ND
120	Neg	ND	ND	ND	ND	ND
137	Neg	ND	ND	ND	ND	ND
151	Neg	ND	ND	ND	ND	ND
165	Neg	ND	ND	ND	ND	ND
179	Neg	ND	ND	ND	ND	ND

Abbreviations: ZIKV = Zika virus; DENV = dengue virus, ND = not done.
